# Externalizing and Internalizing Behaviors in Children with ADHD during Lockdown for COVID-19: The Role of Parental Emotions, Parenting Strategies, and Breaking Lockdown Rules

**DOI:** 10.3390/children9060923

**Published:** 2022-06-20

**Authors:** Maria Grazia Melegari, Pietro Muratori, Oliviero Bruni, Enrica Donolato, Martina Giallonardo, Irene Mammarella

**Affiliations:** 1 Department of Developmental and Social Psychology, Sapienza University, 00185 Rome, Italy; mgraziamelegari@gmail.com (M.G.M.); oliviero.bruni@uniroma1.it (O.B.); 2 IRCCS Fondazione Stella Maris, 56128 Pisa, Italy; 3 Department of Education, University of Oslo, 0317 Oslo, Norway; enrica.donolato@iped.uio.no; 4 Independent Researcher, 00185 Rome, Italy; giallonardomartina@gmail.com; 5 Department of Developmental and Social Psychology, University of Padova, 35122 Padova, Italy; irene.mammarella@unipd.it

**Keywords:** ADHD, parenting, internalizing, externalizing, COVID-19

## Abstract

Lockdown experience for COVID-19 pandemic significantly affected children and adolescents with Attention-Deficit/Hyperactivity Disorder (ADHD) exacerbating or promoting the onset of externalizing and internalizing symptoms. However, few studies have considered how externalizing and internalizing behaviors changed in relation to parental emotions and parenting strategies. In the present study, 992 caregivers of children and adolescents with ADHD from 5 to 18 years were presented with an online survey evaluating youths’ externalizing and internalizing behaviors, their non-compliance with lockdown rules, and parental factors related to parental emotions and parenting strategies. Two hierarchical linear regression models were performed to examine the contribution of children’s non-compliance with lockdown rules, parental emotions, and parenting strategies on children’s externalizing and internalizing behaviors. Results revealed that externalizing behaviors were higher in children and adolescents with ADHD non-compliant with lockdown rules. Moreover, positive parenting strategies moderated the relationship between non-compliance with lockdown rules and externalizing behaviors. Differently, higher internalizing behaviors were observed in children with ADHD who had parents reporting more negative emotions and positive parenting strategies. In this case, parents’ negative emotions had a moderator effect in the association between internalizing behaviors and non-compliance to lockdown measures. The clinical implications of these two different patterns of relations are discussed.

## 1. Introduction

At the end of December 2019, the World Health Organization was informed about a cluster of pneumonia cases caused by a novel coronavirus, the severe acute coronavirus syndrome 2 (SARS-CoV-2). By 11 March 2020, the Coronavirus Disease 2019 (COVID-19) was officially declared a pandemic. Governments worldwide had to take extreme measures to slow down the spread of COVID-19 and try to avert its gravest consequences as much as possible. Suddenly, millions of citizens had their habits and lives disrupted. For instance, in Italy, on 9 March 2020, a national lockdown was imposed, restricting the movements of the population, and mandating the temporary closure of non-essential businesses. Italy was the first country to enact a national lockdown, but most world countries applied similar restrictions in the subsequent months.

Children and adolescents have been among those most severely affected by these necessary radical changes [1]. All face-to-face classes were suspended and gradually offered again by distance education systems; sports facilities were shut down; more importantly, all educational and therapeutic services for children with special needs were temporarily closed. The lockdown drastically reduced youths’ contact with relatives and friends, impoverishing their relational and affective lives. These changes, along with the worries and uncertainties caused by the pandemic, undoubtedly affected the mental health of children and adolescents. Consistently, studies have shown that youths experienced higher levels of depressive symptoms, anxiety, inattention, and irritability [1,2], and prolonged isolation could easily have caused frustration and boredom [3].

The pre-existence of health issues in children and adolescents has been detected as a risk factor for the exacerbation and the development of mental health problems during the COVID-19 pandemic [1,4,5]. In this regard, several studies have highlighted that children and adolescents with Attention-Deficit/Hyperactivity Disorder (ADHD) might represent a particularly vulnerable group [6,7]. ADHD is characterized by attention deficits, hyperactivity, and impulsivity, and youths with ADHD are likely to present comorbidity with internalizing and/or externalizing disorders [8] with 67% of young ADHD sufferers having at least two comorbid disorders. Since the comorbid condition is a critical predictor of the persistence of ADHD itself, it represents an important psychological burden for families of young with ADHD that must learn to manage this complex clinical condition in everyday life.

Studies found a worsening in ADHD-related symptoms and well-being, including greater inattention, irritability, hyperactivity, disruptive behaviors, and elevated sadness or depressed mood in children with ADHD during the pandemic [9,10,11]. Adolescents with ADHD also experienced more sleep problems and learning difficulties than their typically developing peers [12]. School closures, the reduction of outdoor time, the increased screen time (e.g., social media, television, videogames), and isolation might have contributed to the symptoms worsening [9]. However, some studies also reported positive effects of the lockdown for some children, including reduced anxiety, probably due to the changes in the school setting and a more flexible schedule [11].

### 1.1. Being a Parent during a Pandemic

Being a parent of a child or adolescent with ADHD can be quite challenging. Parents have to deal with their child’s emotional and behavioral dysregulation (e.g., inattention, hyperactivity, oppositional behavior) on a daily basis, which often highly compromises the family functioning [13]. This is extremely burdensome, and it is not unusual for parents with ADHD children to experience poor well-being, high levels of stress, anxiety, and frustration [14]. Experiencing negative emotions might hinder the parent-child relationship and increase the use of maladaptive parenting practices [15].

With the advent of the COVID-19 pandemic, parents had to face new and unexpected difficulties, especially those with children with neurodevelopmental disorders such as ADHD. The lockdown led to the closure of schools, and parents had to take care of their child schooling and homework. Access to mental health services and face-to-face assistance was not always possible, and parents had to deal with their child’s difficulties without the usual support of professionals. Moreover, children and adolescents with ADHD had more difficulties complying with the COVID-19 prevention recommendations (e.g., social distancing, avoiding touch, washing hands frequently, and wearing a face mask) [16], adding preoccupation to parents and increasing conflicts with their offspring.

In addition to the usual burdens and the new worries caused by the pandemic (e.g., fear of contagion, financial problems), these factors severely affected parents’ mental health and, therefore, altered their parenting practices. Consistently, Shah et al. [9] reported that a relevant percentage of parents experienced elevated levels of irritability and used maladaptive parenting strategies, including verbal abuse, shouting, and recurrent punishments. Similarly, Pecor et al. [17] found a greater decrease in the quality of life of parents with children with neurodevelopmental disorders compared to caregivers of typically developing children (see also [18,19]).

It is noteworthy that positive changes have also been reported across studies. Parents spent more quality time with their children, leading to increased use of positive parenting strategies (e.g., praising) [6,9]. Moreover, the transition to distance learning helped some parents better understand the offspring’s difficulties (e.g., inattention) and their poor academic performance [11]. These findings suggest that a structured and positive parent-child relationship could curb the exacerbation and/or development of new mental health issues in youths with ADHD during the COVID-19 pandemic (see for instance [20]).

### 1.2. The Current Study

The current study aimed to examine whether parental factors and children’s non-compliance to pandemic measures influenced the functioning of children with ADHD in terms of their internalizing and externalizing behaviors. Parents of a large group of children with ADHD were presented with an online survey assessing their children’s externalizing and internalizing behaviors and non-compliance with lockdown rules. In addition, positive and negative parental emotions and parenting strategies elicited by the child’s behaviors were examined. Previous studies investigated the impact of lockdown on other specific domains in children with ADHD [21,22,23], but, to our knowledge, this is the first study focused on parental emotions and strategies and their association with internalizing and externalizing behaviors of their offspring during the lockdown.

Based on recent literature [24], we hypothesized that the levels of youths’ externalizing behaviors would be associated with parents’ high negative emotions and negative parenting strategies and more difficulties complying with lockdown rules. Moreover, we expected that the levels of youths’ internalizing behaviors would be associated with parents’ positive and negative emotions and supportive parenting strategies.

## 2. Materials and Methods

### 2.1. Participants and Procedure

Data reported in this study are part of an anonymous online survey already presented in previous studies [21,22,23]. Synthetically, parents of children and adolescents with ADHD were asked to evaluate the impact of the lockdown on their children’s behaviors and parental factors related to parental emotions and parenting strategies. The online survey was disclosed through the Italian ADHD Family Association website for a limited time (from 4 June to 21 June 2020). Before accessing the survey, caregivers gave consent to participate in the study. To be included in the study, caregivers had to complete all questionnaires regarding the variables of study within the temporal window terms.

A total of 992 caregivers of children and adolescents with ADHD from 5 to 18 years (85.4% boys, M_age_ = 11.52 years, *SD* = 3.17) satisfied the criteria of selection and none of them were excluded from the study. All caregivers had children and adolescents diagnosed with ADHD by a psychiatrist of the Child and Adolescent Mental Health Services. Information on educational level (Primary school: 0.5%; Middle school: 17.9%; High school: 52.6%; Graduate: 29%) and income (Low: 21.8%; Medium: 75.2%; High: 0.3%) was also collected. The sample was representative of the Italian population as it included participants from all regions, 20 metropolitan cities, and 78.3% of the Italian provinces. Nevertheless, the sample covered the North (53.7%), Center (25.8%), and South (13.4%) of Italy, with higher response rates in Lazio, Lombardia, and Veneto regions.

There was no monetary or credit compensation for participating in the study. The study was approved by the Ethics Committee of the Department of Developmental and Social Psychology, Sapienza University of Rome, and was conducted in accordance with the Declaration of Helsinki (October, 2008).

### 2.2. Measures

*Children’s Internalizing and Externalizing Behaviors*. Eleven items that we considered appropriate to evaluate the psychological and behavioral effect of confinement conditions on children and adolescents with ADHD were selected from the validated Italian version of the Child Behavior Checklist (CBCL) 6–18 questionnaire [25]. The items regarded five internalizing (e.g., “Your child shows [sadness, irritability; little enjoy/interests in activities; boredom; anxiety problems]”) and six externalizing behaviors (e.g., “Your child shows [behavioral problems as verbal or/and physical aggression; oppositional-defiant behaviors]”), grouped into the *externalizing* and *internalizing behaviors* subscales. Parents were requested to choose a single response defining the severity degree based on the frequency by which their children/adolescents expressed each behavioral and emotional-mood dimension during the lockdown (1–2 times/week; 3–4 times/week; 5–7 times/week; absent). These frequencies were scored as follows: 0 = answer omitted/absent; 1 = 1–2 times/week; 2 = 3–4 times/week; 3 = 5–7 times/week. The score of the *internalizing* and *externalizing behaviors* subscales was calculated by adding the related items. The internal consistency of the two subscales in the sample of the present study was good for both internalizing (Cronbach’s α = 0.71) and externalizing behaviors (Cronbach’s α = 0.83).

*Parenting strategies.* The questionnaire included 11 parenting strategies derived from the Alabama Parenting Questionnaire (APQ) [26]. The parents were asked to choose the strategies used during the lockdown with their children through a binary scale (e.g., “Yes”/”No”). The strategies described in the questionnaire comprise two subscales: supportive and preventive strategies or *positive strategies* (e.g., “I listen to his/her requests and try to reassure him/her”; “I stay calm and try to find a compromise*”*), and authoritarian strategies or *negative strategies* (e.g., “I scream, I lose control”; “I punish him/her excessively”). Items rated as “Yes” were scored 1, and those rated as “No” were scored 0. The internal consistency in the sample of the present study was adequate for both positive (Cronbach’s α = 0.69) and negative parenting strategies (Cronbach’s α = 0.62).

*Parent’s emotions.* The questionnaire included a list of three *positive* (e.g., “I feel [optimist; confident]”) and 10 *negative emotions* (e.g., “I feel [frustrated, angry, worried]) that the caregivers might feel in response to their child’s behaviors during the lockdown. For each emotion, the caregivers were asked to choose the emotion experience by using a binary answer (e.g., “Yes”/“No”). Items rated as “Yes” were scored 1, and those rated as “No” were scored 0. The internal consistency of the positive and negative parenting emotions in the sample of the present study was Cronbach’s α = 0.48 and Cronbach’s α = 0.77, respectively.

*Children’s non-compliance with lockdown rules*. The questionnaire included a list of 5 behaviors relating to the preventive measures imposed during the lockdown (e.g., “My child leaves the house although it is forbidden”; “My child refuses to use masks and/or gloves”; “My child shows no concern for the consequences of his actions”). The parents were asked to choose the behavior/s that their children showed during the lockdown. When the parents selected a behavior from the list, the item was scored 1 (on the contrary, the item was scored 0). The internal consistency of this questionnaire in the present sample was adequate (Cronbach’s α = 0.60).

### 2.3. Statistical Analysis

Analyses were performed using the R statistical software [27]. First, descriptive statistics were calculated for all variables of interest. Second, scores were residualized by gender to control for this variable. After that, we examined the association between age, externalizing and internalizing behaviors, children’s non-compliance with lockdown rules, parents’ emotions, and parenting strategies (positive and negative) by performing Pearson’s correlation analysis.

Then, hierarchical linear regression models were performed to assess the contribution of children’s non-compliance with lockdown rules, parents’ emotions, and parenting strategies (variables entered as predictors) on children’s externalizing and internalizing behaviors (outcomes). The linear regressions consisted of four steps, with children’s age and parents’ level of education entered in the first model, children’s non-compliance with lockdown rules in the second, parents’ positive and negative emotions in the third, and positive and negative parenting strategies in the fourth. This method allowed us to analyze the predictive role of each set of variables (e.g., children’s non-compliance with lockdown rule, parents’ emotions, and parenting strategies) over and above the effects of the other variables. Finally, relevant interactions (moderation effects) between children’s non-compliance with lockdown rules and other factors (positive and negative parental emotions and parenting strategies) were examined in the final regression models.

## 3. Results

Descriptive statistics and correlations among all measures are presented in Table 1. After residualizing all variables by gender, children’s externalizing and internalizing behaviors were (1) moderately and positively related to parents’ negative emotions and negative parenting strategies, and (2) weakly and positively associated with positive parenting strategies and children’s non-compliance with lockdown rules. This means that the higher the parents’ negative emotions, positive and negative parenting strategies, and children’s non-compliance with lockdown rules, the higher the children’s externalizing and internalizing behaviors. In addition, children’s externalizing and internalizing behaviors were weakly and negatively related to positive parents’ emotions, indicating that low levels of parents’ positive emotions were associated with elevated children’s externalizing and internalizing behaviors.

### 3.1. Regression of Children’s Externalizing Behaviors

We ran a series of linear hierarchical regression models with children’s externalizing behaviors as an outcome. Children’s age and parents’ level of education were entered in the first step, while the other variables were included in three subsequent steps (see Table 2). Taken together, the final model accounted for a moderate portion of variance in externalizing behaviors, *R*^2^ = 0.32, *p* < 0.001, with children’s non-compliance with lockdown rules, Δ*R*^2^ = 0.04, *F*(1, 984) = 48.4, *p* < 0.001, parents’ positive and negative emotions, Δ*R*^2^ = 0.24, *F*(2, 982) = 165.5, *p* < 0.001, and positive and negative parenting strategies, Δ*R*^2^ = 0.02, *F*(2, 980) = 16.6, *p* < 0.001, accounting for a statistically significant portion of the variance. In the final model (Model 4), the effects of children’s non-compliance with lockdown rules, parents’ negative and positive emotions, and positive and negative parenting strategies were statistically significant predictors, indicating that children’s externalizing behaviors were higher in those who showed non-compliance with lockdown rules and had parents experiencing high negative emotions, low positive emotions, and positive and negative parenting strategies.

Interaction effects between children’s non-compliance with lockdown rules and parents’ variables (parental emotions and parenting strategies) were subsequently examined on children’s externalizing behaviors. Interactions regarding children’s non-compliance with lockdown rules × parents’ negative emotions, *F*(1, 979) = 3.48, *p* = 0.062, children’s non-compliance with lockdown rules × positive emotions, *F*(1, 979) = 3.79, *p* = 0.052, and children’s non-compliance with lockdown rules × negative parenting strategies, *F*(1, 979) = 3.09, *p* = 0.079, did not reach statistical significance. When the interaction children’s non-compliance with lockdown rules × positive parenting strategies was evaluated, *F*(1, 979) = 5.60, *p* = 0.018, a statistically significant effect emerged. This indicated that the positive relationship between children’s non-compliance with lockdown rules and externalizing behaviors decreased for children whose parents used more positive parenting strategies.

### 3.2. Regression on Children’s Internalizing Behaviors

The same procedure described above was applied to examine the effects of our variables of interest on children’s internalizing behaviors (see Table 3). The final model (Model 4) accounted for a relatively moderate portion of variance in internalizing behaviors, *R*^2^ = 0.19, *p* < 0.001. Specifically, children’s non-compliance with lockdown rules, Δ*R*^2^ = 0.01, *F*(1, 984) = 9.20, *p* = 0.002, parents’ positive and negative emotions, Δ*R*^2^ = 0.16, *F*(2, 982) = 94.44, *p* < 0.001, and positive and negative parenting strategies, Δ*R*^2^ = 0.01, *F*(2, 980) = 7.02, *p* < 0.001, accounted for a statistically significant portion of the variance. In the final model, parents’ negative emotions and positive parenting strategies were statistically significant predictors, indicating that children’s internalizing behaviors were higher in those who had parents reporting more negative emotions and positive parenting strategies.

Finally, interactions between children’s non-compliance with lockdown rules and other predictors (parental emotions and parenting strategies) were assessed on children’s internalizing behaviors. A statistically significant effect emerged when children’s non-compliance with lockdown rules × parents’ negative emotion interaction was evaluated, *F*(1, 979) = 4.47, *p* = 0.035. This indicated a positive association between children’s non-compliance with lockdown rules and internalizing behaviors when parents reported low negative emotions and an opposite pattern (negative relationship) when parents experienced high negative emotions. However, the interaction between children’s non-compliance with lockdown rules × parents’ positive emotion, *F*(1, 979) = 0.16, *p* = 0.688, was not statistically significant, with the same results for the interaction between children’s non-compliance with lockdown rules × negative parenting strategies, *F*(1, 979) = 0.56, *p* = 0.455, and children’s non-compliance with lockdown rules × positive parenting strategies, *F*(1, 979) = 3.31, *p* = 0.069.

## 4. Discussion

The lockdown caused by the COVID-19 pandemic abruptly stopped social relationships with relatives and friends, disrupted the usual daily activities, and created the conditions for critical changes in lifestyles. These factors caused critical distress in the world population, including children and adolescents with ADHD and their parents [2,10,11,21]. The current study aimed to examine the extent to which parental factors and children’s non-compliance to pandemic rules were related to internalizing and externalizing behaviors in young patients with ADHD.

Our findings showed that parental distress and punitive/authoritarian parenting represent critical factors in exacerbating externalizing behaviors in youth with ADHD. In addition, children and adolescents with higher vulnerability to tolerating constraints and prohibitions required by the pandemic measures reported higher externalizing behaviors. Nevertheless, our findings could also suggest that these factors were associated with children’s externalizing behavioral problems and that preventive measures implemented during the lockdown exacerbated externalizing behaviors, independently of negative parental factors. Consistently with several pre-pandemic studies on ADHD [24,28,29,30], the positive association between negative parental factors and externalizing behaviors seemed related to conflictual models of child-parent interaction. Although this maladaptive interactive circle is not directly caused by pandemic measures, the lockdown promoted the conditions for critical changes in family lifestyle, including, for example, increased occasions for child-parent arguments [21]. Moreover, the lack of a defined pace of time, previously marked by school and other activities away from home, created critical changes in the previous sleep-wake and screen-time exposure habits compared to the pre-pandemic conditions [22]. Structured family habits and routines are protective factors for children’s behavioral adjustment and positive child-parent interactions [24]. Presumably, parents with high negative emotions had more difficulty contrasting the disruption of these routines, making them more likely to engage in harsh-punitive parenting behaviors exacerbating externalizing children’s behaviors. Conversely, parents who showed positive emotions promoted lower children’s externalizing behaviors. Less obvious is the negative association between positive parenting and externalizing behaviors. McRae et al. [24] point out that, unlike coercive-authoritarian behaviors, positive parenting strategies require more parents’ conscious effort and self-emotional control skills, often acquired after specific training, and it is also possible that other factors related to pandemic measures could have contributed to the quality of parenting strategies. In particular, the interruption of support by trainers, especially for parents who had difficulties accessing the internet, and the missing support of structured contexts (e.g., school), and the disruption of previously established routines, could have weakened parents’ confidence in/or interrupted the continuity of these parenting practices.

Consistently, moderator analysis showed that in parents using positive parenting strategies for managing child discomfort, the positive association between non-compliance with lockdown rules and externalizing behaviors decreased. This finding suggested that children who were more reactive to lockdown rules and exhibited higher externalizing behaviors could benefit from parents adopting positive parenting strategies. In contrast, we did not find a significant association between non-compliance to lockdown rules and other variables of interest on internalizing behaviors. At the same time, we found a significant moderation effect between children’s non-compliance with lockdown rules and parental negative emotions on internalizing behaviors. It is worth noting that these factors could also reflect bi-directional associations with children’s externalizing and internalizing behaviors. Altogether, our findings outlined different risk factors for externalizing and internalizing behaviors in youths with ADHD consistent with distinct phenotypes reported in the literature [31,32,33]. Youths with ADHD and high levels of internalizing behaviors are characterized by elevated irritability but not extreme behavioral reactivity and frustration of externalizing children, which probably makes parents less likely to engage in harsh parenting behaviors. Nevertheless, these children are more vulnerable to stressful events leading them to seek more parental emotional support than children with externalizing behaviors.

The pandemic experience has represented a strong stressful factor among adults [34,35,36] and youths with ADHD [10,23]. Parents with greater vulnerability to distress might have difficulty emotively supporting their children in coping with this experience and modulating their affective states and behaviors. Consistently, parental negative emotions moderated the association between non-compliance to pandemic measures and internalizing behaviors. Lower negative emotions were related to lower internalizing behaviors and higher compliance with lockdown rules. The opposite results were found in parents with high negative emotions: children with ADHD showed higher internalizing problems and non-compliance with lockdown rules. The positive association between positive parenting and children’s internalizing behaviors seemed counterintuitive and discordant with previous literature [37,38]. However, these results should be interpreted within the pandemic context: Italy has been the first European country to be affected by the pandemic, and often contradictory information increased uncertainty about the timing of return to normal activities. This made it very difficult, even for parents with a tendency to use supportive parenting, to convey clear messages of reassurance to their children. Finally, based on the different associations between parental negative and positive emotions and internalizing behaviors, the results suggest that parental negative emotions could be targeted in order to reduce internalizing behaviors in youths with ADHD [39].

The present study has some limitations that need to be duly acknowledged. First, we did not involve a control group, which would have provided further information based on the comparison of respective models of association. However, it is worth noting that some studies have already examined the effects of the lockdown measures in children with developmental disorders compared to typically developing children [19]. Second, we focused exclusively on parents’ emotions and behaviors but other variables (e.g., sleep) could have had a role in accounting for the relationship between non-compliance behaviors during the lockdown and children and adolescents with ADHD internalizing-externalizing problems.

The strength of this study is to provide a comprehensive picture of factors at play in parents/children with ADHD relationships during the pandemic lockdown. We defined different association networks and tested the moderator effects in two phenotypes (e.g., internalizing and externalizing behaviors) in a large sample of children and adolescents with ADHD. These findings could provide relevant information for targeted interventions, mainly for children and adolescents with ADHD who show critical vulnerabilities after restoring normal activities.

## Figures and Tables

**Table 1:** Descriptive statistics (means and standard deviations) and correlations (after residualizing by gender).

	1	2	3	4	5	6	7	8
1. Age in months	1							
2. Children’s externalizing behaviors	−0.105 **	1						
3. Children’s internalizing behaviors	−0.080 *	0.592 **	1					
4. Children’s non-compliance with lockdown rules	0.034	0.205 **	0.087 **	1				
5. Parents’ positive emotions	−0.045	−0.140 **	−0.058	−0.100 **	1			
6. Parents’ negative emotions	−0.063 *	0.517 **	0.415 **	0.153 **	−0.100 **	1		
7. Positive parenting strategies	−0.166 **	0.159 **	0.179 **	−0.023	0.325 **	0.193 **	1	
8. Negative parenting strategies	−0.107 **	0.396 **	0.247 **	0.103 **	−0.093 **	0.544 **	0.087 **	1
M	138.29	7.68	3.64	0.40	0.66	2.94	2.88	1.45
*SD*	38.04	5.34	2.97	0.82	0.86	2.42	1.79	1.37

Note. ** p < 0.01; * p < 0.05.

**Table 2:** Summary of hierarchical regression analysis on children’s externalizing behaviors.

	*b*	SE	*p*	*R* ^2^
Model 1				0.02
Children’s age in months	−0.02	0.00	**<0.001**	
Level of education (high school vs. middle school)	0.86	0.46	0.061	
Level of education (university vs. middle school)	0.56	0.50	0.270	
Model 2				0.06
Children’s age in months	−0.02	0.00	**<0.001**	
Level of education (high school vs. middle school)	0.91	0.45	**0.042**	
Level of education (university vs. middle school)	0.66	0.49	0.178	
Children’s non-compliance with lockdown rules	1.41	0.20	**<0.001**	
Model 3				0.30
Children’s age in months	−0.01	0.00	**0.002**	
Level of education (high school vs. middle school)	0.54	0.39	0.167	
Level of education (university vs. middle school)	0.37	0.43	0.388	
Children’s non-compliance with lockdown rules	0.86	0.18	**<0.001**	
Parents’ positive emotions	−0.53	0.17	**0.002**	
Parents’ negative emotions	1.06	0.06	**<0.001**	
Model 4				0.32
Children’s age in months	−0.01	0.01	**0.029**	
Level of education (high school vs. middle school)	0.34	0.38	0.375	
Level of education (university vs. middle school)	0.09	0.42	0.841	
Children’s non-compliance with lockdown rules	0.85	0.18	**<0.001**	
Parents’ positive emotions	−0.70	0.18	**<0.001**	
Parents’ negative emotions	0.83	0.07	**<0.001**	
Positive parenting strategies	0.30	0.09	**0.001**	
Negative parenting strategies	0.58	0.12	**<0.001**	

Note. Bold values indicate statistical significance.

**Table 3:** Summary of hierarchical regression analysis on children’s internalizing behaviors.

	*b*	SE	*p*	*R* ^2^
Model 1				0.01
Children’s age in months	−0.01	0.00	**0.010**	
Level of education (high school vs. middle school)	0.31	0.25	0.225	
Level of education (university vs. middle school)	0.18	0.28	0.533	
Model 2				0.02
Children’s age in months	−0.01	0.00	**0.007**	
Level of education (high school vs. middle school)	0.32	0.25	0.205	
Level of education (university vs. middle school)	0.20	0.28	0.471	
Children’s non-compliance with lockdown rules	0.35	0.12	**0.002**	
Model 3				0.18
Children’s age in months	0.00	0.00	**0.048**	
Level of education (high school vs. middle school)	0.14	0.23	0.560	
Level of education (university vs. middle school)	0.04	0.26	0.883	
Children’s non-compliance with lockdown rules	0.11	0.11	0.307	
Parents’ positive emotions	−0.07	0.10	0.522	
Parents’ negative emotions	0.49	0.04	**<0.001**	
Model 4				0.19
Children’s age in months	0.01	0.00	0.181	
Level of education (high school vs. middle school)	0.05	0.23	0.843	
Level of education (university vs. middle school)	−0.05	0.26	0.842	
Children’s non-compliance with lockdown rules	0.12	0.11	0.282	
Parents’ positive emotions	−0.20	0.11	0.066	
Parents’ negative emotions	0.45	0.04	**<0.001**	
Positive parenting strategies	0.20	0.05	**<0.001**	
Negative parenting strategies	0.05	0.08	0.490	

Note. Bold values indicate statistical significance.

## Data Availability

Data are available from M.G.M. upon reasonable request.
